# Selection of genes for gene-environment interaction studies: a candidate pathway-based strategy using asthma as an example

**DOI:** 10.1186/1476-069X-12-56

**Published:** 2013-07-03

**Authors:** Marta Rava, Ismaïl Ahmed, Florence Demenais, Margaux Sanchez, Pascale Tubert-Bitter, Rachel Nadif

**Affiliations:** 1 Inserm, Centre for research in Epidemiology and Population Health (CESP), U1018, Respiratory and Environmental Epidemiology Team, F-94807, Paris, Villejuif, France; 2 University Paris-Sud, UMRS 1018, F-94807, Paris, Villejuif, France; 3 Inserm, Centre for research in Epidemiology and Population Health (CESP), U1018, Biostatistics Team, F-94807, Paris, Villejuif, France; 4 Inserm, U946, F-75010, Paris, France; 5 Institut Universitaire d’Hématologie, University Paris Diderot, Sorbonne Paris Cité, F-75007, Paris, France

**Keywords:** Gene by environment interactions, Oxidative stress, Smoking, Pathway-based gene selection

## Abstract

**Background:**

The identification of gene by environment (GxE) interactions has emerged as a challenging but essential task to fully understand the complex mechanism underlying multifactorial diseases. Until now, GxE interactions have been investigated by candidate approaches examining a small number of genes, or agnostically at the genome wide level.

**Presentation of the hypothesis:**

In this paper, we propose a gene selection strategy for investigation of gene-environment interactions. This strategy integrates the information on biological processes shared by genes, the canonical pathways to which they belong and the biological knowledge related to the environment in the gene selection process. It relies on both bioinformatics resources and biological expertise.

**Testing the hypothesis:**

We illustrate our strategy by considering asthma, tobacco smoke as the environmental exposure, and genes sharing the same biological function of “response to oxidative stress”. Our filtering strategy leads to a list of 28 pathways involving 182 genes for further GxE investigation.

**Implications of the hypothesis:**

By integrating the environment into the gene selection process, we expect that our strategy will improve the ability to identify the joint effects and interactions of environmental and genetic factors in disease.

## Background

Until recently, gene by environment (GxE) interaction studies were performed by means of candidate approaches including only a small number of genes. Gene selection in candidate studies relies on 1) known functions of gene sets sharing biological processes, and/or functionally interacting within biological networks; or 2) the mode of action of the environmental factors through relevant pathways in which genes are involved [1]. With the advent of high-throughput genotyping technologies, GxE interactions are starting to be explored at the genome wide level but this approach involves the following difficulties: 1) the heterogeneity of environmental exposures; 2) the “agnostic” nature of the genome-wide approach, which does not make use of prior knowledge on biological processes and/or pathways; and 3) the requirement of stringent thresholds to declare an GxE interaction significant because of the very large number of statistical tests conducted [2].

In this scenario, the classical candidate gene approach can be extended to the selection of large sets of genes. In this paper, we propose a strategy for obtaining a large gene set that integrates the information on biological processes shared by genes, the canonical pathways to which they belong and the biological knowledge related to the environmental exposure studied in the gene selection process.

### The asthma example

Asthma is a complex heterogeneous multifactorial disorder resulting from genetic and environmental factors [3] and whose etiology remains poorly understood. The increase in asthma prevalence in recent decades has led to extensive research regarding the environmental determinants that may have changed over the last 30 years. There have also been considerable efforts to characterize the genetic determinants of asthma, including candidate gene studies, genome-wide linkage screens followed by positional cloning studies and more recently genome-wide association studies (GWAS) [4]. Although these studies have been successful in identifying novel loci, the genetic factors identified explain only a small part of the genetic component of asthma. One of the reasons is that many genetic factors are likely to be involved in the development, the activity and the severity of asthma. Furthermore, they act primarily through complex mechanisms that involve interactions with environmental factors, or with other genes through pathways or networks. The effect of such genetic factors may be missed if their interactions with the environment are not taken into account, or if genes are considered alone, regardless of the biological functions they shared or the pathways they are involved in [5]. Overall, understanding the mechanisms through which genes and the environment interact represents one of the major challenges for pulmonary researchers. The first Genome-Wide Environment Interaction Study (GWEIS) in asthma [6] identified no statistically significant interaction at the genome-wide level, not even with Single Nucleotide Polymorphisms (SNPs), which were shown to interact with the environment in previous candidate studies.

In response to environmental exposures, adaptive responses for protection against environmental toxic insults are activated through metabolic pathways. Among the several metabolic pathways that could be investigated in asthma, the response to oxidative stress is of major interest: the amount of biological evidence of the role of oxidative stress in asthma is increasing [7], and tobacco smoke is related to oxidative stress. Tobacco smoke is also a risk factor for asthma. Active smoking has been found to be associated with the incidence of asthma during adolescence in a dose-dependent manner [8] and with asthma severity in asthmatic cases [9]. Regular smoking was associated with increased risk of new-onset asthma among adolescents in a prospective cohort study [10], and active smoking has a deleterious role on asthma [11]. To our knowledge, only one study focused on gene by smoking interactions on asthma in adults by considering 18 key genes involved in the same pathway: the metabolism of xenobiotics. Some of these genes were also involved in the response to oxidative stress, and SNPs in seven of them were significantly associated with the risk of asthma in adult smokers or non-smokers [12].

### Presentation of the hypothesis

In this paper, we propose a strategy for selecting genes to be investigated in GxE interaction studies. This strategy involves the information on biological processes shared by the genes, the canonical pathways to which they belong to and biological knowledge related to the environment into the gene selection process. We hypothesize that this strategy will provide an expanded and enriched biologically plausible list of candidate genes for further GxE studies.

**Figure 1:**
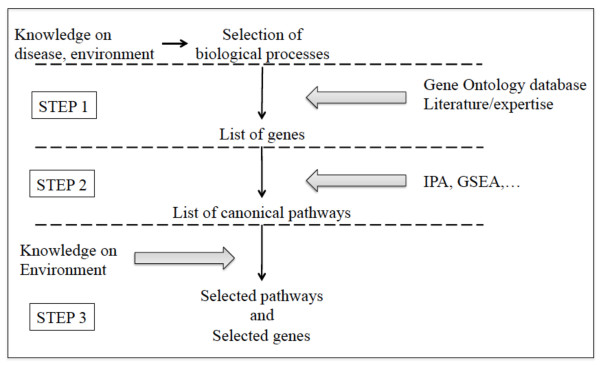
The three-step strategy.

This strategy follows three successive steps (see Figure 1): 1) step 1 (gene selection): selection of a set of genes sharing a biological process known to be related with the outcome or the disease of interest, 2) step 2 (pathway enrichment): selection of physically and/or chemically related gene pathways that are enriched in genes belonging to the gene set selected in step 1. Among the pathways that constitute a biological process, we considered the signaling and/or metabolic pathways, also known as canonical pathways, which better suit the subsequent environmental integration step, and 3) step 3 (environment integration): selection of canonical pathways known to be potentially related to the environmental factor of interest among the pathways selected in step 2. The final set of genes includes the genes selected in step 1 that belong to the canonical pathways selected in step 3. Note that step 3 critically relies on the user’s own expertise.

### Testing the hypothesis

To illustrate our strategy, we consider asthma exposure to tobacco smoke as the environmental factor, and the genes involved in the response to oxidative stress.

### Step 1 (gene selection)

The set of genes was obtained from the Gene Ontology (GO) database (Gene Ontology Consortium [13,14]), as described in the online tutorial [see Additional file 1]. The GO project is a bioinformatics initiative that aims at standardizing the representation of genes and gene product attributes across species and databases. The project provides a controlled vocabulary of terms for describing gene product characteristics and gene product annotation data, as well as tools to access and process this data. We used the term “*response to oxidative stress*” (GO:0006979) which encompasses gene products that are involved in any process that results in a change in state or activity of a cell or an organism (in terms of movement, secretion, enzyme production, gene expression, etc.) as a result of oxidative stress, a state often resulting from exposure to high levels of reactive oxygen species, e.g. superoxide anions, hydrogen peroxide, and hydroxyl radicals. We obtained a set of 387 genes, including all genes previously investigated in candidate GxE interaction studies in respiratory epidemiology such as *MPO*, *CAT*, *GCLM*, *GCLC*, *GSTP1*, *NQO1*[15-21], and some genes in the study by Polonikov *et al*. [12]. We further enlarged the gene set by using our own expertise, GWAS literature reviews, and biological studies [22-26]. A total of 411 genes were then considered for the next step.

### Step 2 (pathway enrichment)

This step consists in identifying canonical pathways that contain a statistically significant excess of genes from the set of 411 genes selected in step 1. This pathway analysis can be conducted by using several tools such as Ingenuity Pathway Analysis (IPA, [27]) or Gene Set Enrichment Analysis (GSEA [28,29]). These software solutions differ in terms of the biological databases they rely on (KEGG, Biocarta, Reactome, Pubmed, STRING…) and the methods used to assess the statistical significance of the pathways.

All gene symbols were recognized by IPA but not by GSEA (390 out of 411). IPA gave 277 canonical pathways that contained at least 5 of the set of 411 genes selected in step 1 and which were significantly enriched in these genes (p < 0.05). IPA P-values for pathway enrichment testing were obtained with Fisher’s exact tests, with a Benjamini–Hochberg correction for multiple testing determined by the ratio of the number of genes from the gene set to the total number of genes in the pathways from the IPA library. GSEA provided no more than the top 100 canonical pathways (p < 1.06 10^-12^). Comparing the results provided by both software packages is difficult as the names of the pathways and the genes involved in them are not standardized. Therefore, we decided to perform the third step with the largest list of pathways and genes *i*.*e*. the 277 pathways obtained from IPA.

### Step 3 (environment integration)

Based on our own expertise, we selected the canonical pathways identified at step 2 that are involved in tobacco smoke metabolism, thus allowing the step 1-gene set to be filtered. Among the 277 canonical pathways identified in step 2, we selected 28 of them (pathway enrichment *P*-values ranging from 2.63x10^-2^ to 1.58x10^-31^) [see Additional file 2: Table S1 and Table S2]. These 28 pathways included from 5 up to 47 genes (15–20 genes on average), 61% of them being involved in more than one pathway. Two hundred and twenty-nine genes from the initial set of 411 genes did not map to any of the selected pathways and were dropped, leading to a final set of 182 genes (Table 1).

**Table 1:** Distribution of the 182 genes by canonical pathways involved in the tobacco smoke metabolism

**Canonical pathways**	P-value*	**N of genes**
NRF2-mediated Oxidative Stress Response	1.58E-31	46
Glutathione Redox Reactions I	1.26E-25	16
Xenobiotic Metabolism Signaling	6.31E-22	44
Aryl Hydrocarbon Receptor Signaling	3.98E-21	32
Mitochondrial Dysfunction	6.31E-19	30
Glutathione-mediated Detoxification	2.00E-18	15
Production of Nitric Oxide and Reactive Oxygen Species in Macrophages	3.98E-16	31
Acute Phase Response Signaling	2.51E-14	28
Antioxidant Action of Vitamin C	1.58E-12	20
IL-8 Signaling	1.26E-11	26
Apoptosis Signaling	2.95E-09	16
Superpathway of Citrulline Metabolism	4.07E-08	7
Superoxide Radicals Degradation	1.05E-07	5
IL-6 Signaling	1.74E-07	16
iNOS Signaling	2.00E-07	10
VEGF Signaling	1.29E-06	13
fMLP Signaling in Neutrophils	1.95E-06	14
Chemokine Signaling	3.02E-06	11
VEGF Family Ligand-Receptor Interactions	5.50E-05	10
NF-KB Signaling	6.31E-05	15
CCR5 Signaling in Macrophages	1.10E-04	9
IL-17A Signaling in Airway Cells	3.89E-04	8
Nucleotide Excision Repair Pathway	4.07E-04	6
IL-1 Signaling	1.10E-03	9
Nicotine Degradation II	2.82E-03	6
Nicotine Degradation III	7.08E-03	5
CCR3 Signaling in Eosinophils	1.51E-02	8
eNOS Signaling	2.63E-02	8

*P-values for pathway enrichment testing as calculated by IPA.

### Implications of the hypothesis

The candidate pathway-based strategy described here was able to select a large number of candidate genes to be tested for interaction with tobacco on asthma. This filtering strategy exploits recent developments in bioinformatics resources that are originally combined with the literature and our own expertise on the metabolism of compounds related to a given environmental factor. This filtering strategy could be applied to other environmental factors related to oxidative stress and asthma, such as outdoor air pollutants or the metabolism of cleaning agents. Together with an expanded and enriched list of candidate genes, the interest of such an approach is also dependent on accurate assessment of environmental exposure. Interestingly, the same list of genes can be used for GxE studies on other diseases characterized by oxidative stress and tobacco smoke, such as lung cancer. By appropriately integrating the knowledge of the environmental factor into the gene selection, we expect that the strategy proposed here will improve the ability to identify the joint effects and interactions of environmental and genetic factors, and will contribute to a better understanding of the etiology of complex diseases.

## Abbreviations

GxE: Gene by environment; GO: Gene ontology; GSEA: Gene set enrichment analysis; GWAS: Genome-wide association studies; GWEIS: Genome-wide environment interaction study; IPA: Ingenuity pathway analysis; SNP: Single nucleotide polymorphism.

## Competing interests

The authors declare that they have no competing interests.

## Authors’ contributions

RN reviewed the literature, designed and developed the strategy, selected the genes and pathways and drafted the manuscript. MR reviewed the literature, participated in the gene-selection process and drafted and revised the manuscript. FD helped to develop the strategy and revised the manuscript critically for important intellectual content. MS participated in data acquisition and revised the manuscript. PTB took part in the development of the strategy and revised critically the manuscript. IA participated in the gene selection process, helped to draft the manuscript and revised critically the manuscript. All authors read and approved the final manuscript.

## Supplementary Material

Additional file 1 Tutorial: Tutorial on how to extract genes from Gene Ontology. Click here for file

Additional file 2: Table S1 List of the 182 genes selected using the pathway-based filtering strategy. **Table S2.** List of the 28 pathways and the relevant genes selected using the pathway-based filtering strategy. Click here for file
