# Supplementary material for: Selection of genes for gene-environment interaction studies: a candidate pathway-based strategy using asthma as an example
Source: Environ Health. 2013 Jul 3;12:56. doi: 10.1186/1476-069X-12-56 (PMC3708788; doi:10.1186/1476-069X-12-56)
Supplement: Additional file 1 — Tutorial: Tutorial on how to extract genes from Gene Ontology. [file 1476-069X-12-56-S1.docx]

**Supplementary file, Tutorial**

Go to <http://amigo.geneontology.org/cgi-bin/amigo/go.cgi>

Choose “Search” and the corresponding page, select “GO terms”.


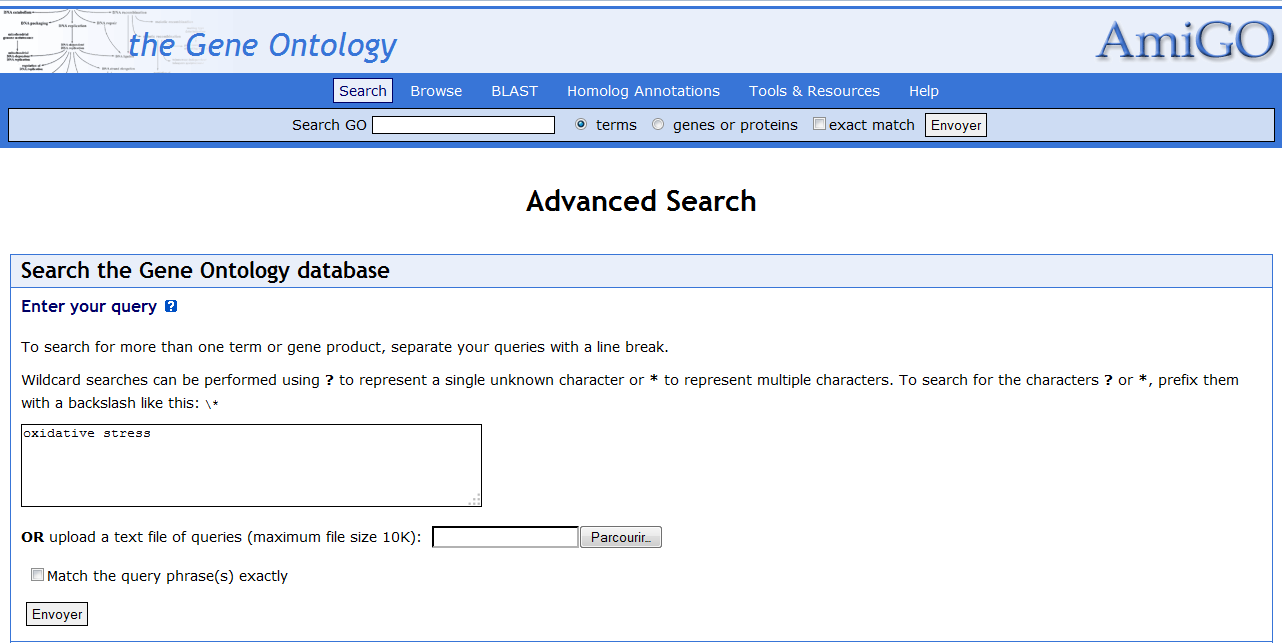
 “Search type”: leave the default options

“Filter results”:

- “Filter by ontology: Select “biological process”
- “Filter Gene Products” - Species: select “Homo sapiens”
- " Filter Gene Products by Associations, Evidence Code": Select all

**--> 22 results for oxidative stress in terms fields term accession, term name and synonyms as follows (May 13rd, 2013):**


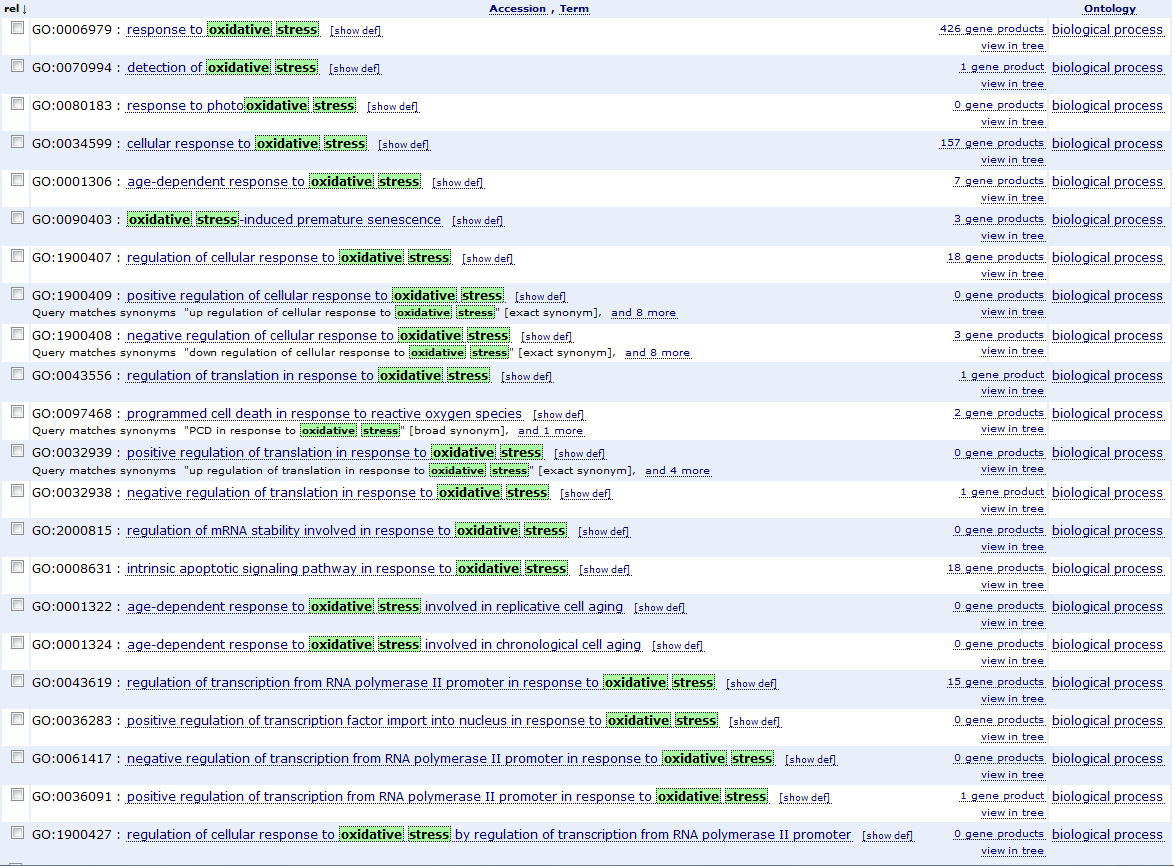


To obtain the tree browser, click on "view in tree" for the 426 genes, and open the node corresponding to GO:0006979


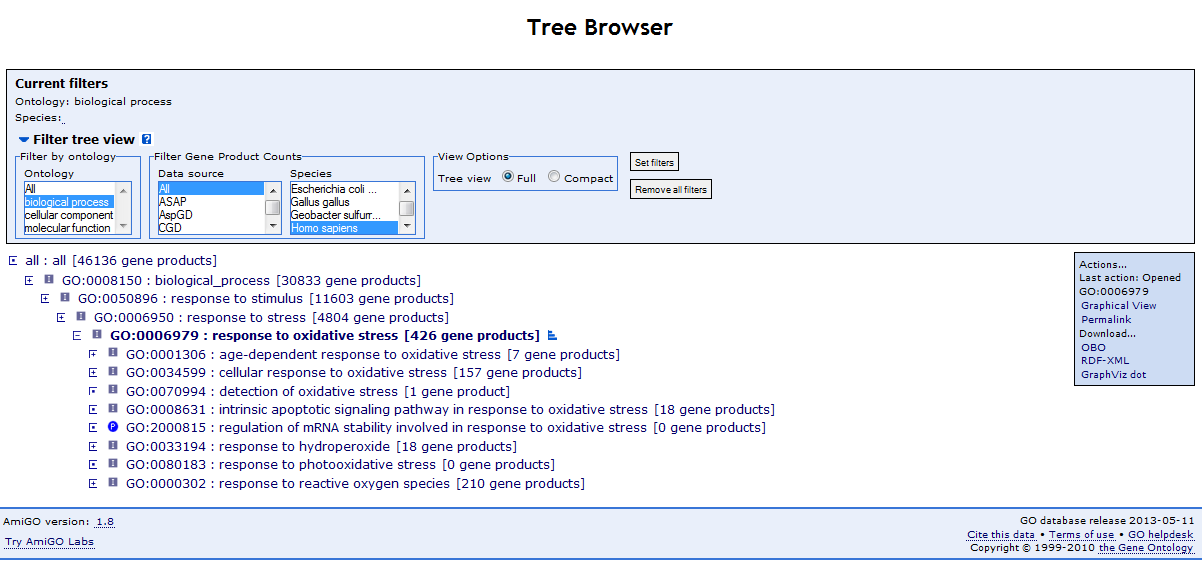


Click to the bar chart to obtain gene product annotation as below:


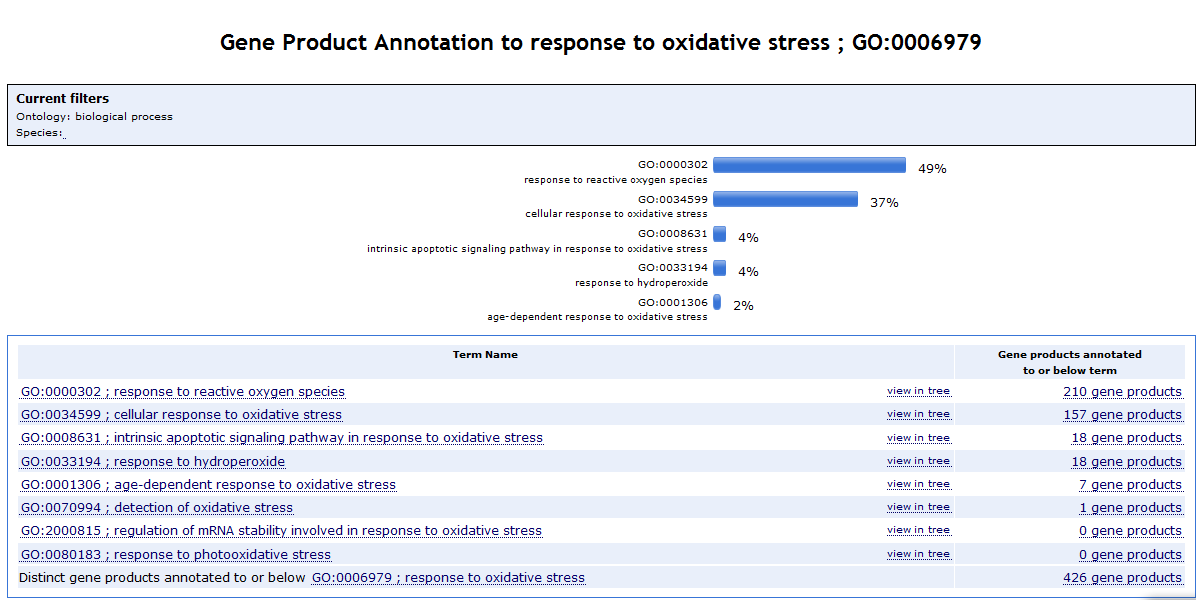


Then extract the 426 genes.
