# Supplementary material for: Selection of genes for gene-environment interaction studies: a candidate pathway-based strategy using asthma as an example
Source: Environ Health. 2013 Jul 3;12:56. doi: 10.1186/1476-069X-12-56 (PMC3708788; doi:10.1186/1476-069X-12-56)
Supplement: Additional file 2: Table S1 — List of the 182 genes selected using the pathway-based filtering strategy. Table S2. List of the 28 pathways and the relevant genes selected using the pathway-based filtering strategy. [file 1476-069X-12-56-S2.docx]

Table S1 List of the 182 genes selected using the pathway-based filtering strategy

| **Symbol** | **Full name** |
| --- | --- |
| ABCC1 | ATP-binding cassette, sub-family C (CFTR/MRP), member 1 |
| AGT | Angiotensinogen |
| AKR1A1 | aldo-keto reductase family 1 |
| AKR7A2 | aldo-keto reductase family 7 |
| AKR7A3 | aldo-keto reductase family 7 |
| ALB | albumin |
| ALDH3B1 | aldehyde dehydrogenase 3 family |
| AOX1 | aldehyde oxidase 1 |
| APOA4 | Apolipoprotein A-IV |
| APOD | apolipoprotein D |
| APOE | Apolipoprotein E |
| APP | amyloid beta (A4) precursor protein |
| AQP1 | Aquaporin-1 |
| ARG1 | arginase, liver |
| ARG2 | arginase, type II |
| ARNT | Aryl hydrocarbon receptor nuclear translocator |
| BAK1 | BCL2-antagonist/killer 1 |
| BCL2 | Apoptosis regulator Bcl-2 |
| BCL2L1 | Bcl-2-like protein 1 |
| BIRC3 | baculoviral IAP repeat containing 3 |
| CAMK2G | calcium/calmodulin-dependent protein kinase II gamma |
| CASP3 | caspase 3, apoptosis-related cysteine peptidase |
| CASP6 | Caspase-6 |
| **CAT** | **Catalase** |
| CCL5 | C-C motif chemokine 5 |
| CDK1 | cyclin-dependent kinase 1 |
| CDK2 | cyclin-dependent kinase 2 |
| CDKN1A | Cyclin-dependent kinase inhibitor 1 |
| CHRNA4 | Neuronal acetylcholine receptor subunit alpha-4 |
| CHUK | Inhibitor of nuclear factor kappa-B kinase subunit alpha |
| CLU | Clusterin |
| COL1A1 | Collagen alpha-1(I) chain |
| COX1 | mitochondrially encoded cytochrome c oxidase I |
| CP | ceruloplasmin (ferroxidase) |
| CYBA | cytochrome b-245, alpha polypeptide |
| CYBB | Cytochrome b-245 heavy chain |
| CYP1A1* | Cytochrome P450 1A1 |
| CYP1A2* | Cytochrome P450 1A2 |
| CYP2E1* | cytochrome P450 |
| DIABLO | diablo |
| EGFR | Epidermal growth factor receptor |
| EP300 | Histone acetyltransferase p300 |
| EPHX1* | epoxide hydrolase 1, microsomal (xenobiotic) |
| ERCC1 | DNA excision repair protein ERCC-1 |
| ERCC2 | TFIIH basal transcription factor complex helicase XPD subunit |
| ERCC3 | TFIIH basal transcription factor complex helicase XPB subunit |
| ERCC6 | DNA excision repair protein ERCC-6 |
| ERCC8 | DNA excision repair protein ERCC-8 |
| F2 | coagulation factor II |
| FMO2 | Dimethylaniline monooxygenase [N-oxide-forming] 2 |
| FOS | Proto-oncogene c-Fos |
| FOSL1 | Fos-related antigen 1 |
| FOXO1 | forkhead box O1 |
| **GCLC** | **Glutamate--cysteine ligase catalytic subunit** |
| **GCLM** | **Glutamate--cysteine ligase regulatory subunit** |
| GLRX | glutaredoxin |
| GLRX2 | Glutaredoxin-2, mitochondrial |
| GLS2 | glutaminase 2 |
| GNAO1 | Guanine nucleotide-binding protein G(o) subunit alpha |
| GPX1 | Glutathione peroxidase 1 |
| GPX2 | Glutathione peroxidase 2 |
| GPX3 | Glutathione peroxidase 3 |
| GPX4 | Phospholipid hydroperoxide glutathione peroxidase, mitochondrial |
| GPX5 | Epididymal secretory glutathione peroxidase |
| GPX6 | Glutathione peroxidase 6 |
| GPX7 | Glutathione peroxidase 7 |
| GPX8 | Probable glutathione peroxidase 8 |
| GRB2 | Growth factor receptor-bound protein 2 |
| GSR | glutathione reductase |
| GSTK1 | glutathione S-transferase kappa 1 |
| GSTM1* | glutathione S-transferase M1 |
| GSTM2 | glutathione S-transferase mu 2 |
| GSTM3 | glutathione S-transferase mu 3 |
| GSTM4 | glutathione S-transferase mu 4 |
| GSTM5 | glutathione S-transferase mu 5 |
| GSTO1 | glutathione S-transferase omega 1 |
| GSTO2 | glutathione S-transferase omega 2 |
| **GSTP1*** | **glutathione S-transferase pi** |
| GSTT1* | glutathione S-transferase theta 1 |
| GSTT2 | glutathione S-transferase theta 2 |
| GSTT2B | glutathione S-transferase theta 2B |
| GSTZ1 | glutathione transferase zeta 1 |
| HIF1A | Hypoxia-inducible factor 1-alpha |
| HMOX1 | Heme oxygenase 1 |
| HMOX2 | Heme oxygenase 2 |
| HNF1A | Hepatocyte nuclear factor 1-alpha |
| HP | Haptoglobin |
| HSPA1A | heat shock 70kDa protein 1A |
| HSPA1B | heat shock 70kDa protein 1B |
| HSPA1L | heat shock 70kDa protein 1-like |
| IL18 | interleukin 18 |
| IL19 | Interleukin-19 |
| IL1A | interleukin 1, alpha |
| IL1B | interleukin 1, beta |
| IL1R1 | interleukin 1 receptor |
| IL6 | interleukin 6 |
| INSR | Insulin receptor |
| JAK2 | Tyrosine-protein kinase JAK2 |
| JUN | Transcription factor AP-1 |
| KEAP1 | kelch-like ECH-associated protein 1 |
| LCK | lymphocyte-specific protein tyrosine kinase |
| LRRK2 | Leucine-rich repeat serine/threonine-protein kinase 2 |
| MAP2K1 | Dual specificity mitogen-activated protein kinase kinase 1 |
| MAP3K5 | mitogen-activated protein kinase kinase kinase 5 |
| MAPK14 | Mitogen-activated protein kinase 14 |
| MBL2 | Mannose-binding protein C |
| MGMT | O-6-methylguanine-DNA methyltransferase |
| MGST1 | microsomal glutathione S-transferase 1 |
| MGST2 | microsomal glutathione S-transferase 2 |
| MGST3 | microsomal glutathione S-transferase 3 |
| MMP9 | matrix metallopeptidase 9 |
| **MPO** | **Myeloperoxidase** |
| NCF1 | Neutrophil cytosol factor 1 |
| NCF2 | Neutrophil cytosol factor 2 |
| ND5 | mitochondrially encoded NADH dehydrogenase 5 |
| NDUFA12 | NADH dehydrogenase [ubiquinone] 1 alpha subcomplex subunit 12 |
| NDUFA13 | NADH dehydrogenase [ubiquinone] 1 alpha subcomplex subunit 13 |
| NDUFA6 | NADH dehydrogenase [ubiquinone] 1 alpha subcomplex subunit 6 |
| NDUFB4 | NADH dehydrogenase [ubiquinone] 1 beta subcomplex subunit 4 |
| NDUFS1 | NADH-ubiquinone oxidoreductase 75 kDa subunit, mitochondrial |
| NDUFS2 | NADH dehydrogenase [ubiquinone] iron-sulfur protein 2, mitochondrial |
| NDUFS3 | NADH dehydrogenase [ubiquinone] iron-sulfur protein 3, mitochondrial |
| NDUFS4 | NADH dehydrogenase [ubiquinone] iron-sulfur protein 4, mitochondrial |
| NDUFS8 | NADH dehydrogenase [ubiquinone] iron-sulfur protein 8, mitochondrial |
| NFE2L2 | nuclear factor (erythroid-derived 2)-like 2 |
| NFKB1 | nuclear factor of kappa light polypeptide gene enhancer in B-cells 1 |
| NOS1 | Nitric oxide synthase 1 (neuronal) |
| NOS2 | nitric oxide synthase 2 |
| NOS3 | Nitric oxide synthase 3 (endothelial cell) |
| NOX1 | NADPH oxidase 1 |
| NOX3 | NADPH oxidase 3 |
| NOX4 | NADPH oxidase 4 |
| **NQO1** | **NAD(P)H dehydrogenase [quinone] 1** |
| NQO2 | NAD(P)H dehydrogenase |
| PARK2 | E3 ubiquitin-protein ligase parkin |
| PARK7 | Protein DJ-1 |
| PDGFRB | platelet-derived growth factor receptor |
| PLA2G4A | Cytosolic phospholipase A2 |
| PLA2R1 | phospholipase A2 receptor 1 |
| PLCB1 | 1-phosphatidylinositol-4,5-bisphosphate phosphodiesterase beta-1 |
| PLCG1 | 1-phosphatidylinositol-4,5-bisphosphate phosphodiesterase gamma-1 |
| PLD2 | Phospholipase D2 |
| POR | P450 (cytochrome) oxidoreductase |
| PPARGC1A | peroxisome proliferator-activated receptor gamma |
| PPP2CB | Serine/threonine-protein phosphatase 2A catalytic subunit beta isoform |
| PRDX1 | Peroxiredoxin-1 |
| PRDX3 | Thioredoxin-dependent peroxide reductase, mitochondrial |
| PRDX5 | Peroxiredoxin-5, mitochondrial |
| PRDX6 | Peroxiredoxin-6 |
| PRKAA1 | protein kinase, AMP-activated |
| PRKCA | Protein kinase C alpha type |
| PRKCD | protein kinase C |
| PRKD1 | protein kinase D1 |
| PRODH | proline dehydrogenase (oxidase) 1 |
| PSEN1 | Presenilin-1 |
| PTGS2 | Prostaglandin G/H synthase 2 |
| PTK2B | Protein-tyrosine kinase 2-beta |
| PXN | Paxillin |
| RAC1 | Ras-related C3 botulinum toxin substrate 1 |
| RAC2 | Ras-related C3 botulinum toxin substrate 2 |
| RELA | Transcription factor p65 |
| RHOB | ras homolog family member B |
| RIPK1 | Receptor-interacting serine/threonine-protein kinase 1 |
| SERPINE1 | Plasminogen activator inhibitor 1 |
| SHC1 | SHC (Src homology 2 domain containing) transforming protein 1 |
| SLC23A2 | Solute carrier family 23 member 2 |
| SNCA | Alpha-synuclein |
| SOD1 | Superoxide dismutase [Cu-Zn] |
| SOD2 | Superoxide dismutase [Mn], mitochondrial |
| SOD3 | superoxide dismutase 3 |
| STAT1 | Signal transducer and activator of transcription 1-alpha/beta |
| TGFBR2 | TGF-beta receptor type-2 |
| TLR4 | Toll-like receptor 4 |
| TP53 | Cellular tumor antigen p53 |
| TXN | Thioredoxin |
| TXN2 | Thioredoxin, mitochondrial |
| TXNDC2 | thioredoxin domain containing 2 |
| TXNRD1 | thioredoxin reductase 1 |
| TXNRD2 | Thioredoxin reductase 2, mitochondrial |
| UCP2 | Mitochondrial uncoupling protein 2 |
| XDH | xanthine dehydrogenase |
| XPA | DNA repair protein complementing XP-A cells |

Genes in bold are those previously investigated in candidate GxE interaction studies in respiratory epidemiology

*Genes studied in the paper by Polonikov et al.

**Table S2. List of the 28 pathways and the relevant genes selected using the pathway-based filtering strategy**

| Pathway | Genes |
| --- | --- |
| NRF2-mediated Oxidative Stress Response | AKR7A2, AKR7A3, GSTM5, PRDX1, NQO2, GCLC, MAP3K5, SOD3, EP300, GSTT1, HMOX1, GSTT2, GSTT2B, AKR1A1, GSTM2, SOD2, JUN, KEAP1, ABCC1, GSTM4, FOSL1, GCLM, TXN, NFE2L2, MAP2K1, PRKD1, PRKCA, GSTK1, GSTM1, MGST1, SOD1, GSTM3, NQO1, TXNRD1, GSTO1, GSR, FOS, MAPK14, MGST2, PRKCD, CAT, GPX2, AOX1, GSTO2, GSTP1, MGST3, EPHX1 |
| Glutathione Redox Reactions I | MGST1, GPX1, GPX5, GPX6, GPX7, PRDX6, GSTZ1, GSR, GPX3, GSTT1, MGST2, GPX2, GPX8, GPX4, MGST3, GSTK1 |
| Xenobiotic Metabolism Signaling | RELA, IL1A, GSTM5, NQO2, GCLC, MAP3K5, IL6, NFKB1, SOD3, ARNT, EP300, HMOX1, GSTT2, GSTT2B, GSTT1, CYP1A2, GSTM2, KEAP1, GSTM4, NOS2, NFE2L2, MAP2K1, PRKD1, GSTK1, PRKCA, GSTM1, MGST1, CYP1A1, FMO2, MGMT, GSTM3, NQO1, GSTO1, PPP2CB, MAPK14, MGST2, PRKCD, CAT, IL1B, ALDH3B1, GSTO2, GSTP1, MGST3, PPARGC1A, CAMK2G |
| Aryl Hydrocarbon Receptor Signaling | RELA, IL1A, GSTM5, NQO2, IL6, NFKB1, EP300, ARNT, GSTT2, GSTT2B, GSTT1, JUN, GSTM2, CYP1A2, GSTM4, NFE2L2, GSTK1, TP53, GSTM1, CYP1A1, MGST1, GSTM3, NQO1, GSTO1, FOS, MGST2, CDKN1A, IL1B, ALDH3B1, GSTO2, GSTP1, CDK2, MGST3 |
| Mitochondrial Dysfunction | PRDX5, XDH, NDUFB4, NDUFS1, SOD2, PARK7, NDUFS2, GPX4, PARK2, NDUFS4, COX1, UCP2, CASP3, GLRX2, LRRK2, NDUFS3, GPX7, NDUFA13, APP, GSR, ND5, PRDX3, NDUFS8, TXN2, NDUFA6, CAT, NDUFA12, TXNRD2, SNCA, PSEN1 |
| Glutathione-mediated Detoxification | GSTM1, MGST1, GSTM5, GSTM3, GSTO1, GSTZ1, GSTT2, GSTT2B, GSTT1, GSTM2, MGST2, GSTM4, GSTO2, GSTP1, MGST3, GSTK1 |
| Production of Nitric Oxide and Reactive Oxygen Species in Macrophages | APOE, RELA, APOA4, CLU, ARG2, JAK2, MAP3K5, NFKB1, JUN, RHOB, CYBA, CYBB, CHUK, NOS2, STAT1, MAP2K1, PRKD1, PRKCA, RAC1, PLCG1, MPO, FOS, TLR4, PPP2CB, NCF1, ALB, MAPK14, PRKCD, NCF2, CAT, APOD |
| Acute Phase Response Signaling | RELA, IL1A, CP, JAK2, MAP3K5, IL6, NFKB1, HNF1A, F2, SHC1, HMOX1, MBL2, SOD2, JUN, CHUK, SERPINE1, MAP2K1, AGT, GRB2, IL1R1, HMOX2, FOS, HP, ALB, IL18, RIPK1, MAPK14, IL1B |
| Antioxidant Action of Vitamin C | RELA, TXNDC2, PLD2, PLA2R1, PLCG1, JAK2, NFKB1, GLRX, PRDX6, GSTO1, TXNRD1, PLA2G4A, HMOX1, SLC23A2, MAPK14, PLCB1, CHUK, TXN, GSTO2, TXNRD2 |
| IL-8 Signaling | RELA, RAC2, PLD2, PTK2B, NOX3, NFKB1, BCL2, HMOX1, JUN, RHOB, CYBB, CHUK, MAP2K1, PRKD1, EGFR, PRKCA, NOX1, NOX4, RAC1, MPO, BCL2L1, FOS, PRKCD, NCF2, PTGS2, MMP9 |
| Apoptosis Signaling | TP53, RELA, CASP3, PLCG1, MAP3K5, NFKB1, BAK1, CDK1, BCL2, CASP6, BCL2L1, CHUK, DIABLO, MAP2K1, BIRC3, PRKCA |
| Superpathway of Citrulline Metabolism | NOS1, PRODH, ARG2, NOS3, NOS2, GLS2, ARG1 |
| Superoxide Radicals Degradation | SOD1, SOD2, CAT, NQO1, SOD3 |
| IL-6 Signaling | RELA, IL1A, GRB2, IL6, IL1R1, JAK2, NFKB1, FOS, COL1A1, SHC1, IL18, JUN, MAPK14, IL1B, CHUK, MAP2K1 |
| iNOS Signaling | TLR4, RELA, FOS, JUN, MAPK14, CHUK, JAK2, STAT1, NFKB1, NOS2 |
| VEGF Signaling | PXN, PTK2B, GRB2, PLCG1, HIF1A, NOS3, ARNT, BCL2, SHC1, BCL2L1, FOXO1, MAP2K1, PRKCA |
| fMLP Signaling in Neutrophils | NOX1, RELA, NOX4, NOX3, RAC1, NFKB1, NCF1, PRKCD, NCF2, CYBB, PLCB1, MAP2K1, PRKD1, PRKCA |
| Chemokine Signaling | NOX1, FOS, JUN, MAPK14, PTK2B, PLCG1, PLCB1, CCL5, MAP2K1, PRKCA, CAMK2G |
| VEGF Family Ligand-Receptor Interactions | SHC1, PLA2G4A, FOS, GRB2, PRKCD, PLCG1, NOS3, MAP2K1, PRKD1, PRKCA |
| NF-KB Signaling | RELA, IL1A, IL1R1, NFKB1, EP300, TGFBR2, TLR4, LCK, IL18, RIPK1, IL1B, CHUK, INSR, EGFR, PDGFRB |
| CCR5 Signaling in Macrophages | FOS, JUN, MAPK14, PTK2B, PRKCD, PLCG1, CCL5, PRKD1, PRKCA |
| IL-17A Signaling in Airway Cells | RELA, IL19, MAPK14, CHUK, JAK2, IL6, NFKB1, MAP2K1 |
| Nucleotide Excision Repair Pathway | ERCC8, ERCC1, ERCC6, ERCC3, ERCC2, XPA |
| IL-1 Signaling | RELA, FOS, IL1A, JUN, MAPK14, GNAO1, IL1R1, CHUK, NFKB1 |
| Nicotine Degradation II | POR, CYP1A1, CYP1A2, FMO2, CYP2E1, AOX1 |
| Nicotine Degradation III | POR, CYP1A1, CYP1A2, CYP2E1, AOX1 |
| CCR3 Signaling in Eosinophils | PLA2G4A, MAPK14, PRKCD, RAC1, PLCB1, MAP2K1, PRKD1, PRKCA |
| eNOS Signaling | CHRNA4, CASP3, HSPA1A, HSPA1B, PRKAA1, PLCG1, AQP1, NOS3, HSPA1L |
